# Warning displays may function as honest signals of toxicity

**DOI:** 10.1098/rspb.2008.1407

**Published:** 2008-11-18

**Authors:** Jonathan D. Blount, Michael P. Speed, Graeme D. Ruxton, Philip A. Stephens

**Affiliations:** 1 Centre for Ecology and Conservation, School of Biosciences, University of Exeter Cornwall Campus, Penryn TR10 9EZ, UK; 2 School of Biological Sciences, Bioscience Building, University of Liverpool Liverpool L69 7ZB, UK; 3 Division of Environmental and Evolutionary Biology, Institute of Biomedical and Life Sciences, University of Glasgow Glasgow G12 8QQ, UK; 4 School of Biological and Biomedical Sciences, University of Durham South Road, Durham DH1 3LE, UK

**Keywords:** aposematism, handicap signal, toxicity, trade-off

## Abstract

Many prey species use colourful ‘aposematic’ signalling to advertise the fact that they are toxic. Some recent studies have shown that the brightness of aposematic displays correlates positively with the strength of toxicity, suggesting that aposematic displays are a form of handicap signal, the conspicuousness of which reliably indicates the level of toxicity. The theoretical consensus in the literature is, however, at odds with this finding. It is commonly assumed that the most toxic prey should have less bright advertisements because they have better chances of surviving attacks and can therefore reduce the costs incurred by signalling. Using a novel theoretical model, we show that aposematic signals can indeed function as handicaps. To generate this prediction, we make a key assumption that the expression of bright displays and the storage of anti-predator toxins compete for resources within prey individuals. One shared currency is energy. However, competition for antioxidant molecules, which serve dual roles as pigments and in protecting prey against oxidative stress when they accumulate toxins, provides a specific candidate resource that could explain signal honesty. Thus, contrary to the prevailing theoretical orthodoxy, warning displays may in fact be honest signals of the level of (rather than simply the existence of) toxicity.

## 1. Introduction

Prey often arm themselves with a repellent defence, such as a toxin, and a bright signal that warns predators of the danger. This phenomenon, known as aposematism, is widely observed across taxa and habitats. It is commonly seen, for example, in insects (e.g. bees and wasps), molluscs (e.g. nudibranchs), reptiles (e.g. coral snakes), amphibians (e.g. dendrobatid frogs), fishes (e.g. puffer fish) and mammals (e.g. skunks). Bright aposematic displays seem to be reliably associated with toxicity because the cost of being conspicuous to predators can only be borne by well-defended individuals (Sherratt 2002). In this sense, warning displays are believed to be qualitatively honest.

However, there has been speculation that warning displays may also be ‘quantitatively honest’ handicap signals, such that the brightness of an aposematic display increases with the toxicity of the prey using the conspicuous advertisement. A positive correlation between signal brightness and toxicity has been reported in an interspecific comparison of one of the most notoriously toxic groups of animals—the dendrobatid (poison) frogs (Summers & Clough 2001)—and recently also in an intraspecific study of Asian ladybird beetles (*Harmonia axyridis*; Bezzerides *et al*. 2007). Paradoxically, however, existing theories of warning signals predict the opposite: that the most toxic prey should have the least bright aposematic advertisements because they are better able to survive attacks and can therefore reduce costs incurred by signalling (Leimar *et al*. 1986; Speed & Ruxton 2005). Consistent with this prediction, warning coloration and toxicity have been shown to correlate negatively across *Epipedobates* species of poison frogs (Darst *et al*. 2006), while there is no apparent correlation between these different components of aposematic defences across populations of strawberry poison frogs (Daly & Myers 1967). Therefore, existing theory does not provide a coherent explanation for whether or how warning coloration and toxicity should correlate.

Here, we show, using a novel theoretical model, that quantitatively reliable aposematic signalling can be predicted when it is assumed that the expression of bright displays and the storage of anti-predator toxins compete for resources within prey individuals. We argue that such competition is likely to be commonplace. One shared currency is energy. However, competition for antioxidant molecules provides a specific candidate resource that could explain signal honesty. Pigment molecules are well known to have antioxidant properties (Olson & Owens 1998; von Schantz *et al*. 1999; McGraw 2005; Griffith *et al*. 2006); antioxidants are also likely to be required to prevent prey damaging themselves when they sequester, biosynthesize and store toxins (Ahmad 1992). When resources are abundant and not limiting, however, our model predicts a negative correlation between warning coloration and toxicity in agreement with earlier theoretical work (Leimar *et al*. 1986; Speed & Ruxton 2007). We first describe our model, which enables a prediction of handicap signalling in aposematism, and then discuss the physiological mechanisms within prey that could render warning displays honest.

## 2. Material and methods

### (a) Modelling scenario

Since we are interested in explaining the optimal conspicuousness of aposematic species, we limit our consideration to prey for which some kind of warning display is optimal (rather than, for example, pure crypsis). We use a deterministic, evolutionary simulation model, and assume that individuals acquire resources from their environment, which they must divide between the storage of defensive toxins and aposematic signalling. Each individual in the population has access to a given level of resource for their lifetime. Prey that have access to the same level of resource are considered members of a ‘resource group’. Prey cannot predict which resource group they will be allocated to and so we assume that they evolve a set of alternative optimal strategies, which are expressed conditional on finding themselves in a given resource group. For example, a prey that finds itself with a very high resource level may have a different optimal strategy for dividing its resource to one that has a low resource level. When our simulations evolve to equilibrium, prey choose from a set of strategies (one for each resource state), which maximizes their fitness for each resource level. At the start of the simulations, members of each resource group show a full range of possible allocation strategies. Over evolutionary time, strategies that do not maximize fitness are selected against, so that the endpoint yields a set of alternative optimal allocations for each resource state.

Resource allocation strategies are game theoretic, in the sense that the optimal strategies of individuals with a given resource depend on the choices of prey with other resource levels. For example, the rate at which an individual is encountered by a predator increases with its level of signalling relative to other prey in its own and in other resource groups. Furthermore, the probability that such an encounter leads to an attack decreases with the mean level of investment in defences across the whole population. Finally, the probability that an attack on an individual causes its death declines (multiplicatively) with that individual's level of investment in defences and with the extent of their signalling.

### (b) Modelling details

To evaluate the circumstances in which warning displays could act as reliable signals of the strength of defence, we constructed a model in which prey within a single population partition a limited resource between toxicity and signalling. We assume that there are five equally abundant resource levels available (denoted *R*(*i*), where *i*=1, 2, …, 5; though the number of resource states can be increased without affecting the qualitative nature of our predictions), and that prey are assigned randomly to one of these ‘resource groups’ for their lifetime. The prey must decide how to allocate its resource optimally between aposematic display (*A*) and secondary defence (*D*), assumed to be an internally stored toxin.

Individuals within the prey population allocate the *R*(*i*) resources available to them according to a heritable trait, *A* (0≤*A*≤1). *A* determines the proportion of available resources that are allocated to aposematism. The complement of *A* is *D* (*D*=1−*A*), the proportion of available resources that are allocated to secondary defences.

Thus, for an individual with access to resources *R*(*i*), trait *A* dictates investment in both aposematism and secondary defences (because these two uses compete for the resource). Here, we model *A* on a discrete grid to the nearest percentage point (*A*=0.00, 0.01, 0.02, …, 1.00). The prey population is modelled deterministically by considering the proportion *f*(*i*,*A*) of individuals in any resource group with any given trait value, where

(2.1) $$\sum_{i=1}^{5}\sum_{A=0.00}^{1.00}f(i,A)=1.$$

The total population is *N*_0_ and the total number of individuals in any resource group is *N*_*i*_=0.2*N*_0_.

Simulations are initiated with a uniform distribution of individuals with all possible trait values within each resource group (i.e. *f*(*i*, *A*) is initially identical for all *i* and *A*). Frequencies of individuals with different trait types are then assumed to evolve in response to selection imposed by predation. Specifically, predation imposes differential survival, *S*(*i*, *A*), on individuals with different attributes (investment in aposematism and secondary defences), and this affects the relative proportions of different types of individual that are represented in the next generation. Strictly, we assume that survival is the only component of fitness that is affected by an individual's attributes, such that the relative frequency of a given type of individual after survival and breeding is given by

(2.2) $$f^{\prime }(i,A)=\sum_{A^{\prime }=0.00}^{1.00}f(i,A^{\prime }).S(i,A^{\prime }).z,$$

where *z* is given by the indicator function

$$z=\{\begin{array}{cc} 1-\epsilon & A^{\prime }=A\\ \epsilon /100 & A^{\prime }\neq A \end{array}.$$

Equation (2.2) ensures that, at each generation, there is some low level of mutation, *ε*. Mutation from any trait value to any other trait value is equally likely. Thus, every trait value loses *ε* of its potential representation in the next generation to mutation, and gains *ε*/100 of the potential representation of every other trait value within that resource group. This guarantees that solutions to the model are evolutionarily stable by ensuring that every trait type always has the opportunity to invade from rare. As stated, equation (2.2) gives the relative representation of different traits in the next generation. This is rescaled to ensure that the total frequencies over all resource groups sum to unity (equation (2.1)), using

$$f^{\prime \prime }(i,A)=\frac{0.2f^{\prime }(i,A)}{\sum_{A}f^{\prime }(i,A)}.$$

Survival of individuals in any generation is dependent on their resource group and *A* trait value. Specifically, survival depends on: the rate at which predators are encountered, *r*(*i*,*A*); the probability of attack given an encounter, *p*_1_(*i*,*A*); and the probability of death given an attack, *p*_2_(*i*,*A*). Survival is thus given by

(2.3) $$S(i,A)=e^{-r(i,A).p_{1}(i,A).p_{2}(i,A)}.$$

The rate at which individual prey encounter predators is dependent on their relative conspicuousness. The absolute conspicuousness of any given individual is given by

(2.4) $$c(i,A)=1.5-e^{-\alpha AR(i)},$$

where *α* is a constant that scales the rate at which conspicuousness increases with investment in aposematism. This gives a value between 0.5 (for zero investment in aposematism) and a maximum of 1.5 for higher investment in aposematism. Higher values of *α* lead to a more rapid increase in conspicuousness with increasing coloration. The mean absolute conspicuousness across the whole prey population is

$$\bar{c}=\sum_{i}\sum_{A=0.00}^{1.00}c(i,A).f(i,A),$$

and the trait-specific encounter rates are given by

(2.5) $$r(i,A)=c(i,A)/\bar{c}.$$

The probability that a prey individual, once encountered, is attacked, is assumed to depend on the mean level of secondary defences in the population as a whole, D^*^. This is given by

$$D^{*}=\sum_{i}\sum_{A=0.00}^{1.00}\left(1-A)R(i).f(i,A\right).$$

Consequently, our basic formulation for the probability of attack is

(2.6) $$p_{1}(i,A)=0.01+0.99{\text{e}}^{-0.1D^{*}},$$

where 0.1 scales the exponent. In this formulation, the probability of attack is the same for all prey individuals, is bounded between 0.01 and 1.00 (to ensure that no type of individual is completely invulnerable to attack) and increases as population mean toxicity decreases.

We assume that the predator is prepared by evolution to handle brightly coloured prey with care. This is a major evolutionary reason that toxic prey use aposematic displays and it is well supported in the empirical literature (Gamberale-Stille & Tullberg 1999; Gamberale-Stille & Guilford 2004). We also assume that secondary defences can increase the probability of survival at this stage (Wiklund & Järvi 1982; Skelhorn & Rowe 2006*a*,*b*). Thus, the probability that a prey individual dies as a result of an attack is assumed to decrease as a result of increased investment in both aposematism and secondary defences. Our basic formulation is

(2.7a) $$p_{2}(i,A)=0.01+0.99{\text{e}}^{-0.1AR(i).(1-A)R(i)},$$

where the first term in the exponent is investment in aposematism and the second term is investment in secondary defence. Alternative formulations for *p*_*1*_ and *p*_*2*_, and variation in the values of *ϵ*, have made little difference to our qualitative findings and are described in the electronic supplementary material. The only alternative formulation that changes the main result is if we assume that predators make separate, non-interacting assessments of aposematic displays and toxins when determining how hard to attack the prey, that is

(2.7b) $$p_{2}(i,A)=0.01+0.99{\text{e}}^{-0.1[AR(i)+(1-A)R(i)]}=0.01+0.99{\text{e}}^{-0.1R(i)}.$$

Clearly, the relative scaling of conspicuousness (a negative consequence of coloration; equations (2.4) and (2.5)) and predator caution (a positive consequence of coloration; equations (2.7*a*) or (2.7*b*)) is crucial to the outcome of the model (see further in the electronic supplementary material). These scalings cannot easily be inferred from empirical data and the formulae we use are, thus, to some extent arbitrary. Our intention here is to expose the potential of the mechanism to induce honesty in aposematic displays. In so doing, we highlight the value of further empirical studies to assess the relative scaling of these phenomena.

At the start of a simulation, all possible allocation phenotypes are present in all resource groups and when stability is reached suboptimal allocation strategies are removed from the population. We simulated the evolution of prey populations under different conditions until stability was reached (where stability is defined as the summed absolute magnitudes of changes among frequencies of all trait types were less than 10^−8^ per generation). All results shown reflect these stable solutions. For the levels of mutation used, there was a single optimum value of *A* in each resource group. Variance around that was negligible and so only the mean value of *A* is shown. Unless otherwise stated, we use the values *α*=0.01, *ϵ*=10^−6^ and *R*(5)=10 in our simulations.

## 3. Results

**Figure 1:**
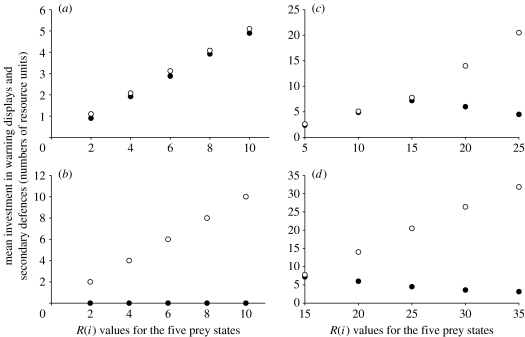
Optimal values of warning displays and secondary defences for a set of resource states. Open circles represent secondary defences (toxicity) and filled circles represent warning displays. *R*1–*R*5 are equally abundant, such that 20% of prey are assigned to one resource group (*α*=0.01). (*a*) Equation (2.7*a*) is employed (in which display and secondary defences interact to protect prey that are being attacked) and resource values between 2 and 10 are used. (*b*) Equation (2.7*b*) is employed (in which display and secondary defences do not interact to protect prey that are being attacked) and resource values between 2 and 10 are used. (*c*) Equation (2.7*a*) is employed and resource values between 5 and 25 are used. The optimal response varies in a non-monotonic manner between resource groups. (*d*) Equation (2.7*a*) is employed and resource values between 15 and 35 are used. The optimal allocation of resources to aposematism now declines monotonically as resource levels increase.

In the first use of the model, we assume that there are five equally abundant resource levels available within a single population (denoted *R*(*i*), where *i*=1, 2, …, 5), and that prey are assigned randomly to one of these for their lifetime. For this environment (resource levels between a value of 0 and 10 resource units), the system evolved to a stable solution where prey individuals with brighter warning signals are indeed those with better defences (figure 1*a*). Here, aposematic signals are quantitatively honest, in the sense that the more toxic prey have the costlier signals. This result is robust to variations in the formulation of the probability of death given detection (see the electronic supplementary material).

There is some empirical support for the prediction of within-population reliable signalling (Bezzerides *et al*. 2007). However, the strongest empirical evidence for reliable signalling in aposematism is found across dendrobatid frog species rather than within a single population (Summers & Clough 2001). It is easy to demonstrate cross-species (or cross-population) signal reliability in our model by simulating a series of populations within which all resource types are of equal value, and then to vary resource values across populations. Considering the resource states independently in this manner did not affect the positive correlation between coloration and toxicity (indeed, the graphs are quantitatively very similar whether we assume that resource variation falls within populations, as in figure 1*a*, or between populations). Hence, signal reliability across the dendrobatid frogs can be explained by our model if the brightest and deadliest species gain access to more of the limiting resource than those that are less bright and less deadly.

We found two situations in which a positive correlation between defence and conspicuousness is not predicted. The first is when predators assess aposematic displays and toxins independently when determining how hard to attack the prey (see equation (2.7*b*) in §2). Then, the optimal strategy for prey is always to invest in toxins and never in aposematic displays (figure 1*b*). Here, a unit of resource spent on displays provides the same survival benefit during an attack as a unit invested in toxins, but displays incur additional costs of conspicuousness and provide a lower net return.

The second situation in which a prediction of signal honesty breaks down is seen when resource availability exceeds some threshold. Our model predicts that, at high resource values, more toxic prey have less bright displays (figure 1*c*,*d*). Our model suggests that when prey have very abundant resources it pays to divert them increasingly into toxins, because a sufficiently toxic prey can protect itself from injury during attacks (equation (2.7*a* and 2.7*b*); §2), even with a low level of aposematic display. Relatively dull coloured but highly toxic prey encounter predators less often and have very high chances of surviving attacks.

By contrast, for prey at the lower end of the resource spectrum, if an individual puts all of its resource into toxins, it will be insufficiently repellent to provide good protection during an attack. When the resource is very limited, signalling brightness therefore increases with toxicity, because the pairing of moderate signal and toxin levels has a disproportionately beneficial effect on prey survival (compared with investing all resources in toxins). Our model therefore incorporates the more conventionally predicted negative correlation between colours and toxins, but predicts that the positive correlation between defence and display occurs when the key resource is limited.

## 4. Discussion

To predict reliable signalling of the level of defence in aposematism, we had to make two essential assumptions. First, predators are sensitive to the combined qualities of toxins and displays when they attack prey. In our model (especially using equation (2.7*a*)), prey must have some non-zero value of both display and toxicity if they are to increase their chances of surviving an attack through aposematic defences. In support of this, there is good empirical evidence that predators seem to be prepared by generations of predator–prey coevolution to handle aposematic prey more carefully during attacks than non-aposematic prey (Wiklund & Järvi 1982; Sherratt 2002; Gamberale-Stille & Guilford 2004; Skelhorn & Rowe 2006*a*,*b*).

The second essential component of the model is that warning coloration and toxicity compete for the same resource. Several recent studies have reported that warning coloration varies among individuals of the same species (de Jong *et al*. 1991; Holloway *et al*. 1995; Bezzerides *et al*. 2007; Sandre *et al*. 2007), and avian predators have been shown to be responsive to such variation, being more wary of more saturated colour signals (Gamberale-Stille & Tullberg 1999). Recently, it has been shown that the extent or intensity of warning coloration can correlate positively with levels of chemical defences, both within species (Bezzerides *et al*. 2007) and across species (Summers & Clough 2001). This empirical evidence points to the possibility that warning displays may be ‘handicap signals’, meaning that they are honest indicators of defensive capability, for which reliability is guaranteed by the high cost of signal production (Zahavi & Zahavi 1997). However, such handicap signalling would require that production of warning colours should ‘use up’ some of the resource that is itself needed to produce chemical defences—it has been difficult to envisage how such specificity between warning colours and chemical defences could exist (Guilford & Dawkins 1993). We suggest that warning coloration and chemical defences could indeed be linked through the competitive use of a shared resource.

Life-history trade-offs have traditionally been considered in terms of energy allocations (Stearns 1992). Indeed, energy has been suggested as a putative limiting factor in the acquisition, biosynthesis or storage of toxins (Holloway *et al*. 1991) and also the costs of warning displays (Srygley 2004), although the latter have received relatively little attention (see review in Ruxton *et al*. 2004). There is little basis to think that energy availability could mediate trade-offs between warning coloration and toxicity. The literature on sexual signalling suggests that while energy may in part limit signal expression by influencing foraging efficiency, trade-offs in the physiological allocation of pigments used in signals also apply (Blount & McGraw 2008). As with sexual signals, aposematic coloration is commonly imparted by pigments including carotenoids, flavonoids, melanins, ommochromes, papiliochromes, pteridines and porphyrins (Needham 1974; Bornefeld & Czygan 1975; Britton *et al*. 1977; Nijhout 1991; Summers *et al*. 2003), all of which have the potential to function as antioxidants *in vivo* (McGraw 2005). Use of antioxidant pigments to impart warning coloration could be costly, and inversely related to the capacity to produce or maintain toxicity, in at least two different ways.

First, use of antioxidants to impart colour could directly trade against their availability to prevent self-damage caused by toxins. Such autotoxicity has been highlighted as a potential cost to chemically defended organisms (Ahmad 1992; Tollrian and Harvell 1999). Many plant allelochemicals are powerful pro-oxidants, which, when ingested, can cause oxidative stress (Ahmad 1992). In laboratory rats, β-carotene (a carotenoid) has been shown to afford protection against oxidative stress induced by monocrotaline (Baybutt & Molteni 1999)—a pyrrolizidine alkaloid commonly used as a chemical defence in Lepidoptera and Coleoptera. Therefore, it has been hypothesized that antioxidants must be accumulated to protect against autotoxicity in chemically defended prey (Ahmad 1992). Second, the sequestration or biosynthesis of toxins and storage facilities, or antioxidant pigments, may itself risk oxidative stress. Here, costs are mediated through high levels of oxidative metabolism and concomitant production of reactive oxygen species (ROS), which can cause serious damage to biomolecules, rather than a lack of energy *per se* (von Schantz *et al*. 1999). For example, isolation of toxins through encapsulation could be costly (Tollrian and Harvell 1999), because encapsulation reactions cause generation of ROS and therefore risk oxidative stress (Ojala *et al*. 2005).

The potential influence of antioxidant availability and oxidative stress on the development of aposematic displays has recently begun to be considered (Ojala *et al*. 2005; Sandre *et al*. 2007). As yet, however, there have been no studies of whether antioxidants may be traded between warning coloration and the production or maintenance of toxicity; some key questions remain unanswered. For example, could trade-offs in antioxidant usage between coloration and toxicity occur where both pigments and toxins are found in the same physical location (e.g. skin cells) in aposematic organisms? This seems possible, because antioxidant pigments (and therefore coloration) will be depleted when such compounds donate themselves as antioxidants. Alternatively, trade-offs in antioxidant allocation to coloration versus antioxidant defence may occur ‘upstream’, if antioxidants are required to protect sensitive tissues from oxidative damage during toxin transport to different body parts. Animals may face foraging constraints for antioxidant molecules themselves (carotenoids and flavonoids) or for specific nutrients such as amino acids required for pigment biosynthesis (melanins, ommochromes, papiliochromes, pteridines and porphyrins; Olson & Owens 1998; Griffith *et al*. 2006). In addition, antioxidants may be rendered limiting for components of aposematic defences if they are required for other body functions such as immune defence (Ojala *et al*. 2005) or reproduction (Sandre *et al*. 2007). We think that the dual role of animal pigments as colourants and antioxidants makes them strong candidate resources for trade-offs between different components of aposematic defence.

## 5. Honesty and dishonesty in the model

We found that, when predators assess aposematic displays and toxicity independently in determining how hard to attack prey, the optimal strategy for prey is always to invest in toxins and never in conspicuousness (figure 1*b*). Here, a unit of resource spent on displays provides the same survival benefit during an attack as a unit invested in toxins, but displays incur additional costs of conspicuousness (i.e. detectability), and provide a lower net return. Given that aposematism is abundant in nature and that, on empirical grounds, predators are unlikely to ignore the toxicity of prey when they attack them (Gamberale-Stille & Guilford 2004; Skelhorn & Rowe 2006*a*,*b*), this scenario seems implausible.

The second situation in which a prediction of signal honesty breaks down is seen when resource availability exceeds some threshold. Our model predicts that, at high resource values, more toxic prey have less conspicuous displays (figure 1*c*,*d*). Here, the result matches the prediction from other theoretical models of aposematism, in which signalling patterns are the inverse of the reliable signalling model (Leimar *et al*. 1986; Speed 2001). If antioxidants are required to enable high levels of toxicity, as we have hypothesized, then highly toxic but relatively drab prey are predicted to use high levels of non-pigment antioxidants (e.g. antioxidant enzymes, vitamin E) or, alternatively, high levels of antioxidant pigments capable of imparting relatively dull coloration such as melanins. In work on poison frog species, Darst *et al*. (2006) found that warning coloration and toxicity were negatively correlated: the most conspicuous species (*Epipedobates bilinguis*) is only moderately toxic, and the most toxic species (*Epipedobates parvulus*) is not the most conspicuous, while a third species (*Epipedobates hahneli*) shows moderate levels of both conspicuousness and toxicity. Captive trials showed that domestic hens were equally averse when presented with highly conspicuous species and highly toxic species of poison frogs, respectively (Darst *et al*. 2006). It therefore seems possible that while a positive correlation between conspicuousness and toxicity may arise during the initial evolution of aposematism (Summers & Clough 2001), these different components of aposematic defences may subsequently become dissociated and independently adjusted as individual species use different combinations to achieve the same effect (Darst *et al*. 2006). The results of our model suggest an alternative potential explanation for why warning coloration and toxicity may correlate negatively. When prey have very abundant resources, it pays to divert them increasingly into toxins, because a sufficiently toxic prey can protect itself from injury during attacks (equation (2.7*a*); §2), even with a low level of aposematic display. Relatively drab but highly toxic prey encounter predators less often and have very high chances of surviving attacks.

It is important to note that for simplicity of presentation, we limit our model to the set of organisms for which aposematism is a beneficial phenotype. Hence, prey in our model that invest little in signalling are not by implication very highly cryptic, they merely have relatively inconspicuous warning displays. It is, in our view, possible that the coloration used for highly cryptic appearances uses resources in the same way as coloration for aposematic display. Hence, it is equally possible to model the optimal investment of toxins and pigments for cryptic prey (and to determine the parameters under which prey choose maximal crypsis without toxicity, or some combination of the two). However, since the focus of our immediate question is signal honesty in aposematic prey, we have omitted this part of the model in this presentation.

## 6. Conclusions

In conclusion, our model ‘squares a circle’ in aposematism research. The theoretical expectation has been that brightness as a general quality can reliably indicate the existence of toxicity, but that within (or between similar) species there should be a negative correlation between the level of display and toxicity (Leimar *et al*. 1986; Speed & Ruxton 2005); a state of ‘quantitative dishonesty’. Rigorously collected datasets show opposing patterns: the most toxic individuals (Bezzerides *et al*. 2007) and species (Summers & Clough 2001) can have the most conspicuous coloration or the least conspicuous coloration (Darst *et al*. 2006). We have demonstrated that, if displays and defences compete for a shared resource, warning signals can indeed be honest handicaps. However, when the availability of the key resource is not limiting, individuals or species should be highly toxic and warning displays dishonest. Our model therefore yields new, testable predictions for the evolution of warning signal diversity.

## Supplementary Material

Robustness considerations Description of tests to show that the model is robust to variation in parameter values and formulation of key equations
