# Supplementary material for: Warning displays may function as honest signals of toxicity
Source: Proc Biol Sci. 2008 Nov 18;276(1658):871–7. doi: 10.1098/rspb.2008.1407 (PMC2664363; doi:10.1098/rspb.2008.1407)
Supplement: Robustness considerations — Description of tests to show that the model is robust to variation in parameter values and formulation of key equations [file rspb20081407s25.doc]

**Supplementary material**

**Robustness considerations**

Our results are qualitatively unaffected by variation in the value of *ε* (between 1x10^-7^ and 1x10^-2^, Figure S1) and in α (values between 10^-3^ and 10^-1^: Figure S2). Nevertheless, altering the value of α does lead to quantitative changes in the outcome (Fig. S2). The reason for this is that, as α changes, so the relative scaling of conspicuousness (the negative consequence of colouration, Eqns. 4 and 5 in main text) and predator caution (a positive consequence of colouration, Eqns. 7a or 7b in main text) also changes. If α is changed sufficiently (e,g, α = 1.0), the observed results do not suggest honest signalling across the range of resource levels modelled. However, our focus in this paper is to consider whether the mechanism that we propose can lead to honesty in aposematic displays. That it can do so suggests that empirical work to quantify the underlying relationships would be highly valuable.

Using the model we found that for the conditions simulated, the system evolved to a stable solution where prey individuals with brighter warning signals are indeed those with better defences (Fig. 1A main paper). Here aposematic signals are quantitatively honest. This general result of a positive correlation between the brightness of aposematic displays and toxicity is robust against variation in assumptions about the precise formulation of the probability of attack given detection. As we use it in the main text, equation 6 is:

$p_{1}(i,A)=0.01+0.99e^{-0.1D*}$ (referred to here as 6a)

If we modified equation 6a so that attack probability decreases with increasing investment in aposematism as well as toxicity, either in a multiplicative relationship

$p_{1}(i,A)=0.01+0.99e^{-0.1D*.AR(i)}$, (6b)

or additively

$p_{1}(i,A)=0.01+0.99e^{-0.1[D*+AR(i)]}$. (6c)

then the prediction of reliable signalling pertains (Fig. S3AB). The only notable difference is that with a multiplicative relationship between A and D (in equation 6b), the optimal behaviour of the prey is to invest more of the common resource in displays than toxins.

When predators make separate assessments of aposematic displays and toxins when determining how hard to attack the prey, that is if we rewrite equation 7a but now prevent A and D interacting such that

$\begin{matrix} p_{2}(i,A)=0.01+0.99e^{-0.1[AR(i)+(1-A)R(i)]} \\ =0.01+0.99e^{-0.1R(i)} \end{matrix}$. (7b)

then the optimal strategy for prey is always to invest in toxins and never in aposematic displays (Figure S3C, using equation 6a). Similarly, if we keep equation 7b, and employ equations 6b or 6c, the optimal response, that prey never invest in toxins, is also improbable.

(a)

(b)

Mean investment in aposematism (filled circles) and secondary defences

(open circles) (numbers of resource units)

(d)

(c)

(e)

(f)

*R*(*i*) values for the five prey states

Figure S1. Effects of mutation rate, *ε*, on outcomes of the simulations. In each case, *R*(*i*) = 2*i*, *α* = 0.01 and probabilities of attack and death are modelled using eqns 6a and 7a. Mutation rates are (a) *ε* = 10^-7^; (b) *ε* = 10^-6^; (c) *ε* = 10^-5^; (d) *ε* = 10^-4^; (e) *ε* = 10^-3^; (f) *ε* = 10^-2^. Clearly, the absolute level of mutation has no effect on the final results. Hereafter, we used a low level of mutation of *ε* = 10^-6^.

(a)

Mean investment in aposematism (filled circles) and secondary defences

(open circles) (numbers of resource units)

(b)

(c)

*R*(*i*) values for the five prey states

Figure S2. Effects of varying the parameter *α*. In each case, *R*(*i*) = 2*i*, *ε* = 10^-6^. and probabilities of attack and death are modelled using eqns 6a and 7a. Conspicuousness scaling is given by (a) *α* = 10^-3^; (b) *α* = 10^-2^; (c) *α* = 10^-1^. Eqn. 4 dictates that conspicuousness increases more rapidly for low *A* and approaches an asymptote at high *A*. However, this relationship is affected by the absolute value of *α*, such that at low *α* the increase in conspicuousness is approximately linear with increasing *A*, whilst at high *α* the increase is much more rapid for low *A* than for high *A*. The consequences of this can be seen from the increasing disparity in investment in secondary defences and aposematism at low *A*, when *α* is increased.

(a)

Mean investment in aposematism (filled circles) and secondary defences

(open circles) (numbers of resource units)

(b)

(c)

*R*(*i*) values for the five prey states

Fig. S3. Effects of formulations for probabilities of attack and of prey death on signalling honesty. All panels show the same situation as in Fig. 1a, except that (a) uses eqn. 6b; (b) uses eqn. 6c; (c) uses eqn. 7b (and 6a).
